# Study on Transorgan Regulation of Intervertebral Disc and Extra-Skeletal Organs Through Exosomes Derived From Bone Marrow Mesenchymal Stem Cells

**DOI:** 10.3389/fcell.2021.741183

**Published:** 2021-09-23

**Authors:** Zhikun Wang, Yangming Wu, Zhonghan Zhao, Chengyi Liu, Lingli Zhang

**Affiliations:** ^1^School of Kinesiology, Shanghai University of Sport, Shanghai, China; ^2^School of Physical Education and Sports Science, South China Normal University, Guangzhou, China

**Keywords:** exosomes, bone marrow mesenchymal stem cells, transorgan regulation, endocrine regulation, intervertebral disc

## Abstract

Exosomes are membranous lipid vesicles fused with intracellular multicellular bodies and then released into the extracellular environment. They contain various bioactive substances, including proteins, mRNA, miRNAs, lncRNAs, circRNAs, lipids, transcription factors, and cytokine receptors. Under certain conditions, bone marrow mesenchymal stem cells (BMSCs) can differentiate into osteoblasts, chondrocytes, adipocytes, and biological functions. This study provides a theoretical basis for the application of exosomes derived from bone marrow mesenchymal stem cells (BMSC-Exos) in osteology, exploring different sources of exosomes to improve bone microenvironment and resist bone metastasis. We also provided new ideas for the prevention and rehabilitation of human diseases by exosomes.

## Introduction

The exosome is a nanoscale membranous vesicle with a double-layer lipid membrane structure. It is spherical or cup-shaped, with a diameter of about 40–100 nm and a density of about 1.13–1.21 g/mL extracellularly secreted by various cells in the body. Exosomes can be used as a medium for communication between cells (Tkach and Thery, 2016). They contain various bioactive substances, including proteins, mRNA, miRNA, lncRNA, lipids, transcription factors, and cytokine receptors. The current general view is that exosomes are originally formed through endocytosis (Tkach and Thery, 2016). Briefly, the cell membrane is internalized to produce the endosome, after which small vesicles are formed inside the endosome by invading certain parts of the endosome membrane. Such an endosome is called a multivesicular body (MVB). Eventually, MVB fuses with the cell membrane and releases endosomal vesicles in the lumen into the extracellular space as exosomes.

Non-specific expression protein found in the exosomes can be used as the recognition of exosomes protein markers, including heat shock protein, tumor susceptibility gene101 (TSG101), four-molecule cross-linked transmembrane protein superfamily (CD9, CD63, CD81, CD82), and ALG-2-interacting protein (Alix) (Tkach and Thery, 2016). Moreover, some specific expression proteins are cell-derived and related to cell signal transduction. There are differences in the expression of these proteins under different physiological and pathological conditions, including osteoclast-derived exosomes containing receptor activator of NF-κB (RANK) and T cell-derived exosomes containing T-cell surface glycoprotein CD3ε chain (CD3) (Zhang et al., 2015). These proteins are very important in the diagnosis of diseases. Besides, exosomes contain nucleic acids, such as DNA, mRNA, miRNA, lncRNA, circRNA, etc., which have an important role in information transmission and regulation of a variety of physiological and pathological processes (Tkach and Thery, 2016). Previous studies have suggested that the proportion of miRNA in exosomes is higher than that in mother cells (Zhang et al., 2015).

The biological composition of exosomes is related to the types of cells, and exosomes secreted by different cells have different biological functions. The lipids, proteins, and nucleic acids carried by exosomes are transported to the recipient cells through endocrine or paracrine transportation to regulate the biological activity of the recipient cells. For example, activated T cells can recruit dendritic cells (DC) derived exosomes containing major histocompatibility complex (MHC) class II and down-regulate the immune response through the interaction of T cells and DC (Nolte-’t Hoen et al., 2009). Exosomes are also involved in cell phenotypic regulation and tissue regeneration. For example, exosomes derived from liver stem cells can promote liver cell regeneration (Herrera et al., 2010). Exosomes, which also have a beneficial role in sepsis by increasing the phagocytosis of apoptotic cells (Miksa et al., 2006), participate in disease transmission and the genesis of tumors. In addition, they can create a favorable microenvironment for tumors, lead to the failure of treatment, and promote the growth and metastasis of tumor cells (Tickner et al., 2014). Moreover, they can induce the differentiation of mesenchymal stem cells (MSCs) into carcinoma-associated fibroblasts (CAFs) (Cho et al., 2012), promote the proliferation and longevity of fibroblasts, weaken the immune response, and then lead to the invasion and metastasis of tumor cells.

In this review, we introduced and discussed the following: (1) The mechanism of exosomes derived from bone marrow mesenchymal stem cells (BMSC-Exos) regulating the proliferation and differentiation of osteoblasts and osteoclasts through mRNA, miRNA, lncRNA, and circRNA; (2) the impact of BMSC-Exos on human organs and their significant therapeutic effects on the motor system, cardiovascular system, and nervous system. If exosomes are used as research targets, they can have a prominent role in intervertebral disc degeneration, tendon regeneration, wound repair, cardiovascular system protection, nerve injury treatment, and clinical disease diagnosis. (3) The regulatory effects of exosomes from extraosseous sources on BMSCs, and the regulatory activity network of exosomes secreted by extraosseous organs and tissues on BMSCs.

## The Structure and Function of Bone Marrow Mesenchymal Stem Cells-Exos

### Characteristics of Bone-Derived Exosomes

The composition of bone-derived exosomes differs from exosomes originated from other sources. During bone reconstruction, bone-derived exosomes may release protein involved in bone formation and factors, such as alkaline protease (ALP), bone morphogenetic protein (BMP), eukaryotic translation initiation factor2 (elF2) and non-collagenous matrix proteins, e.g., osteopontin (OPN), bone sialoprotein (BSP), and osteocalcin (OCN). Osteocyte-related exosomes also contain osteoclast differentiation-related proteins such as the receptor activator of nuclear factor κB–ligand (RANKL) and RANK (Huynh et al., 2016). In addition, they contain miRNAs-related to bone remodeling, such as miR-24, let-7, miR-143-3p, miR-10b-5p, miR-199b, miR-218, and miR-214-3p (Huynh et al., 2016; Li et al., 2016). These miRNAs have a crucial role in bone formation and resorption. Other specific mRNAs found in bone-related exosomes are factor TFIID subunit 7-like (TAF7L), SOX-11 of transcription factors, and lysophosphatidic acid receptor 1 (LPAR1) and zinc finger E-box-binding homeobox 2 (ZEB2) etc., that regulate transcription initiation of transcription (Morhayim et al., 2017). Yet, significant differences in the types of miRNAs in exosomes may be found during osteogenic induction compared with other stages. In addition, age can also affect another important factor of miRNAs in exosomes. Compared with mice of other age groups, the content of miR-96, miR-182, and miR-183 isolated from bone marrow tissue fluid of young mice was significantly higher compared to older mice (Davis et al., 2017). In addition, the release frequency of exosomes is also related to pathological conditions, such as inflammation, hypoxia, and pH changes (Deng et al., 2017).

### The Characteristics and Function of Bone Marrow Mesenchymal Stem Cells-Exos

BMSCs derived from the mesoderm are a kind of stem cell with multi-differentiation potential that has an important role in bone regeneration and repair. BMSCs also have paracrine functions (Eirin et al., 2017). For example, the study found that BMSCs inhibit interleukin-1 β (IL-1β) induced degenerative effects in NP cells by their paracrine activity. Recent studies have found that the paracrine mechanism of BMSCs is closely involved in tissue repair (Fouraschen et al., 2012). At the same time, BMSCs-Exos can alleviate immune rejection and can have a biological function similar to stem cells (Ionescu et al., 2012; Yamaguchi et al., 2015).

BMSC-Exos contains a variety of anti-inflammatory factors and growth factors, such as tumor necrosis factor (TNF)-stimulated gene 6 (TSG-6), transforming growth factor-β (TGF-β), interleukin 10 (IL-10), and tumor necrosis factor-α (TNF-α). Compared with BMSCs, BMSC-Exos is smaller and has no cell activity. BMSC-Exos can act as a nanoscale target carrier, making good use of the enhanced osmotic effect. It can not only penetrate the blood-brain barrier and blood-spinal barrier but also selectively penetrate into the inflammatory tissue sites (van den Boorn et al., 2011; El Andaloussi et al., 2013). Transplantation of BMSC-Exos can promote bone regeneration and repair bone defects, having similar biological functions to BMSCs. BMSC-Exos, which promote bone regeneration by upregulating OCN expression, is associated with the activation of the Wnt/β-catenin pathway. Due to lower immunogenicity and cell activity, they are considered safer than transplanted stem cells (Liu et al., 2015).

BMSC-Exos have an obvious role in the prevention and rehabilitation of diseases of different organs and tissues, which can promote cardiovascular regeneration and inhibit myocardial infarction, help to accelerate tendon regeneration and skin wound healing, nerve repair, and improve the cognitive status of patients (Liu et al., 2015). Also, BMSC-Exos have been shown to be effective in the treatment of liver, lung, kidney, acute injury. Meanwhile, exercise can indirectly activate BMSC-Exos to improve osteoarthritis (OA) (Lai et al., 2016).

**FIGURE 1:**
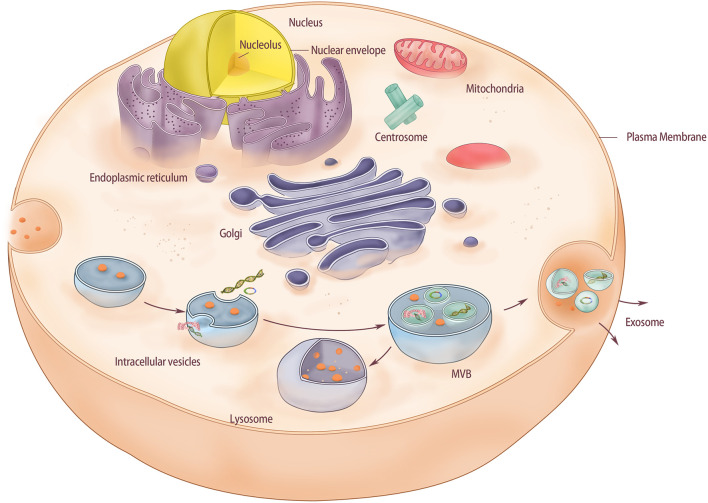
The structure of exosomes derived from bone marrow mesenchymal stem cells. Membrane-driven endocytosis forms intracellular vesicles in the cytoplasm and stores substances from extracellular fluid in the vesicles. As intracellular vesicles mature gradually in the cytoplasm, intracellular vesicles continue to form inside the vesicles. Intracellular vesicles can store substances in the cytoplasm and form MVB. Finally, MVB fused with the cell membrane and released the intracavinal vesicles, which completed the release of exosomes. In this process, if MVB is directly fused with lysosomes in the cytoplasm, polycysts will be degraded.

Compared to BMSCs, BMSC-Exos have certain advantages such as simple extraction method; extracted BMSC-Exos can be stored at –20°C for 6 months by keeping their biological activity (Lai et al., 2016); they possess strong proliferation ability, good anti-pollution, and strong viability, all of which have made them an interesting area of research over the years (Figure 1).

## Bone Marrow Mesenchymal Stem Cells-Exos Transorgan Regulation

### Effects of Bone Marrow Mesenchymal Stem Cells-Exos on Intervertebral Disc

The Intervertebral disc is a structure with hydrodynamic characteristics, composed of three parts: nucleus pulposus, annulus fibrosus, and cartilage plate. The nucleus pulposus is the central part; the annulus fibrosus is the peripheral part that surrounds the nucleus pulposus, while the cartilage plate is the upper and lower part, directly connected with the vertebral bone tissue. Intervertebral discs bear loads in complex ways during daily activities and are usually combined with compression, flexion, and torsion. When the intervertebral disc is under pressure, the pressure per unit area is about 1.5 times that of the external force as the nucleus pulposus can only be slightly compressed. Consequently, the pressure makes the intervertebral disc bulge around, and the peripheral tensile stress is mainly borne by the surrounding annular fibrous ring (Neidlinger-Wilke et al., 2014).

#### Effect of Bone Marrow Mesenchymal Stem Cells-Exos on Nucleus Pulposus Cells of Intervertebral Disc

The main pathological changes of intervertebral disc degeneration (IDD) are nucleus pulposus cells (NPC) reduction and extracellular matrix (ECM) decomposition. The current treatment strategies, including surgical and non-operative treatment, cannot supplement the reduced NP cells or reverse the pathological changes of IDD. Recent studies have found that MSCs can differentiate into NPC under certain conditions and alleviate the progression of IDD (Han et al., 2015; Shim et al., 2016). At present, the commonly used exogenous stem cells for the study and treatment of IDD mainly include bone marrow mesenchymal stromal cells, BMSCs, adipose mesenchymal stem cells, and muscle-derived stem cells, etc. As the most commonly used stem cells in cell therapy and tissue engineering, BMSCs show great advantages in IDD therapy (Feng et al., 2011). Recent studies have shown that BMSC-Exos might also be effective in repairing intervertebral discs degeneration. Cheng et al. (2018) found that BMSC-Exos can inhibit the apoptosis of NPC induced by TNF-α, which may be related to the fact that exosome miR-21 targets phosphatase and tensin homolog (PTEN) into NPC through phosphatidylinositol 3-kinase (PI3K)-serine/threonine-protein kinase (AKT) pathway. Xia found that BMSC-Exos may significantly prevent the occurrence of degeneration by inhibiting the activation of inflammatory mediators and NLRP3 inflammatory bodies in NPC in the rabbit IDD model (Xia et al., 2019). In addition, another study found that exosomes can provide mitochondrial proteins for NPC, and the damaged mitochondria can be repaired by supplementing exosomes (Xia et al., 2019). Matrix metalloproteinase (MMP), especially MMP-1 and MMP-3, is a marker protein of nucleus pulposus cell degeneration that can decompose ECM. Tissue inhibitor of metalloproteinase (TIMP) is a tissue inhibitor protein of MMPs, which can inhibit nucleus pulposus degeneration. By detecting these three marker proteins (Barile et al., 2017), it was found that BMSC-Exos could reduce the expression levels of MMP-1 and MMP-3 in nucleus pulposus cells and increase the expression level of TIMP-1, thus indicating that BMSC-Exos could alleviate the aging of nucleus pulposus cells.

#### Effect of Bone Marrow Mesenchymal Stem Cells-Exos on Annulus Fibrosus Cells of Intervertebral Disc

In the process of intervertebral disc degeneration, the synthesis and distribution of ECM tend to change. The synthesis of type II collagen by nucleus pulposus cells and inner annulus fibrosus cells decreases, while type I collagen increases (Yang and O’Connell, 2017). A large number of intracellular inflammatory factors such as IL-1β and tumor necrosis factor-α (TNF-α) has been found to be released outside the cell in the IDD, resulting in the high expression of MMP and a disintegrin and metalloproteinase with thrombospondin motifs (ADAMTS) enzymes in the ECM (Purmessur et al., 2013; Vo Nam et al., 2013). However, these two kinds of enzymes can destroy the ECM of the intervertebral disc, manifested by the massive loss of proteoglycan and type II collagen in the ECM and the increase of type I collagen content (Weiler et al., 2012). This, in turn, leads to pathological changes such as proliferation and disorder of collagen fibers in the annulus fibrosus of the intervertebral disc. Li et al. obtained exocrine bodies from BMSCs of patients with non-open femoral fractures (Li Z. Q. et al., 2020). Then, IL-1 β and IL-1 β were combined with exosomes to treat annulus fibrosus (AF) cells. He and his team found that BMSC-Exos inhibited IL-1β, induced inflammation, and apoptosis, and promoted the proliferation of AF cells. Further analysis also found that BMSC-Exos may reduce the inflammatory response of annulus fibrosus cells by inhibiting autophagy mediated by PI3K/AKT signal pathway.

**FIGURE 2:**
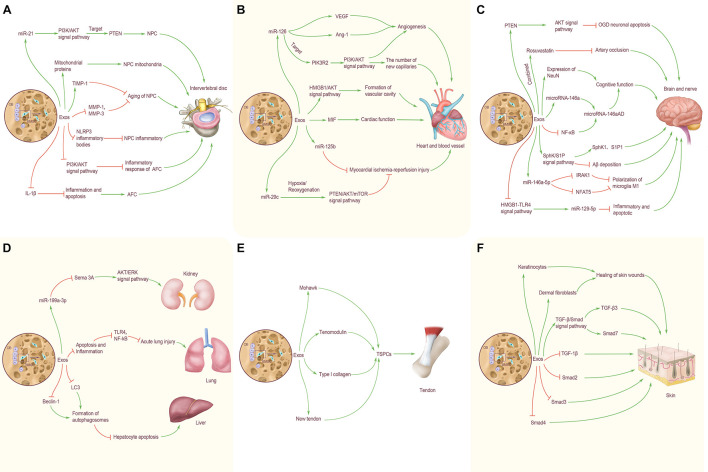
BMSC-Exos transorgan regulation. **(A)** Effects of BMSC-Exos on intervertebral disc. MiR-21 in BMC-Exos inhibits NPC apoptosis by targeting PTEN through PI3K/AKT signaling pathway. BMC-Exos can provide NPC with mitochondrial proteins, and repair damaged mitochondria by supplementing exosomes. BMC-Exos can increase the expression level of TIMP-1 and inhibit the aging of nucleus pulposus cells. BMSC-Exos can reduce the expression levels of MMP-1 and MMP-3 in nucleus pulposus cells and alleviate the aging of nucleus pulposus cells, play an anti-inflammatory role in NP cells by inhibiting the activation of NLRP3 inflammatory bodies and in AFC by inhibiting autophagy mediated by PI3K/AKT signaling pathway. BMSC-Exos inhibited IL-1β induced inflammation and apoptosis, and promoted AFC proliferation. **(B)** Effects of BMSC-Exos on heart and blood vessel. BMSC-Exos miR-126 up-regulated the expression of VEGF and Ang-1 and promoted angiogenesis; activated the PI3K/Akt signaling pathway by targeting PIK3R2, and significantly increased the number of new capillaries on the wound. BMSC-Exos can promote angiogenesis by activating HMGB1/AKT signal pathway. High expression of MIF-BMSC-Exos can better enhance cardiac function; miR-125b carried by BMSC-Exos has a therapeutic effect on myocardial ischemia reperfusion injury. Under hypoxia/reoxygenation conditions, cells can inhibit excessive autophagy through the PTEN/AKT/mTOR signal pathway, and change the expression level ofmiR-29c, so as to reduce myocardial ischemia reperfusion injury. **(C)** Effects of BMSC-Exos on brain and nerve. BMSCs can reduce the apoptosis of OGD neurons and cells by regulating the expression of PTEN and activating the AKT signaling pathway. The combined treatment of BMSC-Exos and Rosuvastatin was more effective in inhibiting arterial occlusion. BMSC-Exos enhanced the expression of NeuN, increased the level of microRNA-146a and decreased the level of NF-κB in astrocytes, thereby increasing the expression of microRNa-146aAD in hippocampus and improving cognitive dysfunction. BMSC-Exos can increase the level of microRNA-146a and decrease the level of NF-κB in astrocytes, thereby increasing the expression of microRNa-146aAD in hippocampus and improving cognitive dysfunction. By activating the SphK/S1P signal pathway, BMSC-Exos enhance the expression of SphK1 and S1P1, and reduce the deposition of Aβ. BMSC-Exos rich in miR-146a-5p can directly down-regulate the expression of IRAK1 and NFAT5, inhibit the polarization of microglia M1, and improve neurological function. BMSC-Exos can exert the anti-inflammatory and anti-apoptotic effects of miR129-5p by inhibiting the activity of HMGB1-TLR4 pathway. **(D)** Effects of BMSC-Exos on lung, liver and kidney. BMSC-Exos can down-regulate the expression of Sema 3A through the delivery of miR-199a-3p to renal cells, thus activating the AKT and ERK signal pathways, protecting the kidney and alleviating kidney damage. BMSC-Exos can reduce the TLR4 and NF- κB by reducing apoptosis and inflammation, inhibit the TLR4/NF-κB, and alleviate acute lung injury caused by ischemia-reperfusion; up-regulate the expression of LC3 and Beclin-1, reduce the apoptosis of hepatocytes after acute liver failure. **(E)** Effects of BMSC-Exos on tendon. BMSC-Exos can enhance the expression of Mohawk, Tenomodulin and type I collagen, as well as the mechanical properties of the new tendon, and promote the proliferation of local TSPC *in vivo*. **(F)** Effects of BMSC-Exos on skin. BMSC-Exos can effectively promote the proliferation of human keratinocytes and dermal fibroblasts and accelerate the healing of skin wounds. BMSC-Exos can also up-regulate the expression of TGF-β3 and Smad7 in the TGF-β/Smad signal pathway, down-regulate the expression of TGF-β1, Smad2, Smad3, and Smad4, and promote skin wound healing.

Previous studies have preliminarily shown the great potential of BMSC-Exos in the treatment of IDD. BMSC-Exos can promote the proliferation of degenerated NPC (Lu et al., 2017). Exosomes derived from NPC can promote BMSCs migration and induce BMSCs to differentiate into NP-like phenotypes. As an important tool for information exchange between BMSCs and NPC, exosomes have multiple functions and advantages. The application or loading of specific genes and drugs alone is a new strategy for acellular therapy of IDD (Lu et al., 2017). Nevertheless, the participation of BMSC-Exos in IDD is mainly concentrated in nucleus pulposus cells, and there is a lack of research on annulus fibrosus cells (Figure 2A).

### Effects of Bone Marrow Mesenchymal Stem Cells-Exos on Brain and Nerve

Stroke can be classified into hemorrhagic stroke and ischemic stroke. Recent studies have found that BMSC-Exos have a positive role in preventing stroke and improving the recovery of neurological function after brain injury. A previous study found that BMSC-Exos rich in miR-146a-5p can directly down-regulate the expression of IRAK1 and NFAT5, inhibit the polarization of microglia M1 after intracerebral hemorrhage, reduce the inflammatory response, and reduce nerve cell apoptosis in male rats with intracerebral hemorrhage, and in turn protect and improve nerve function after cerebral hemorrhage (Duan et al., 2020). Similarly, BMSC-Exos also have a positive role in improving the early brain injury caused by subarachnoid hemorrhage. BMSC-Exos can exert the anti-inflammatory and anti-apoptotic effects of miR-129-5p by inhibiting the activity of the HMGB1-TLR4 pathway, thereby alleviating the early brain injury after subarachnoid hemorrhage (Xiong et al., 2020). Moreover, BMSC-Exos combined with rosuvastatin, a cholesterol-lowering drug, reduced the infarct size in rats with middle cerebral artery occlusion after 7 days compared to the control group treated with BMSC-Exos alone (Safakheil and Safakheil, 2020). The combination of BMSC-Exos and rosuvastatin as a new treatment regimen may have an important role in functional recovery and neuroprotection after ischemic stroke.

In addition, BMSC-Exos also have the special role in improving cognitive function and delaying the onset of Alzheimer’s disease (AD). BMSC-Exos injected into mice can reduce Aβ deposition, enhance the expression of SPHK1 and S1P1, and improve the spatial learning and memory ability by activating the SPHK/S1p signaling pathway. In addition, BMSC-Exos can also inhibit mice’s brain amyloid protein expression in the cortex and hippocampus, enhance the expression of NeuN, and promote the recovery of cognitive function in AD mice (Wang and Yang, 2021). BMSCs were injected into the ventricle and attached to the choroid plexus of the lateral ventricle so that BMSC-Exos could be secreted into the cerebrospinal fluid. When astrocytes ingest BMSC-Exos, they can increase the level of miR-146a in astrocytes and decrease the level of NF-κB, improve the inflammation of astrocytes, and synaptic formation, thus increasing the expression of miR-146aAD in the hippocampus and improving the cognitive impairment in mice (Nakano et al., 2020). To sum up, these data suggest that BMSC-Exos have an important role in treating stroke, promoting the recovery of neurological function, and improving cognitive function. Although there are still many problems to be solved, it is undeniable that BMSC-Exos represent a new treatment direction for the above-mentioned diseases and provide new possibilities for the rehabilitation of patients (Figure 2C).

### Effects of Bone Marrow Mesenchymal Stem Cells-Exos on Heart and Blood Vessel

Over recent years, exosomes have shown a beneficial effect in protecting myocardial cells, inhibiting myocardial infarction, promoting angiogenesis, and maintaining cardiac function. Routine 3D-Exos culture of bone showed that 3D BMSC-Exos could promote proliferation and migration of endothelial cells by activating the HMGB1/AKT pathway, resulting in the formation of the vascular cavity and promoting angiogenesis. 3D BMSC-Exos are expected to become a potential therapeutic method for promoting angiogenesis in the field of human medical research (Gao et al., 2020).

High levels of micro RNA-126 (miR-126), an endothelium-specific miRNA associated with vascular homeostasis and angiogenesis, have been isolated from BMSC-Exos by the hypervelocity centrifugation method. Studies have shown that miR-126 of BMSC-Exos can promote the proliferation, migration, and angiogenesis of human umbilical vein endothelial cells by inducing the expression of vascular endothelial growth factor (VEGF) and angiotensin-1 (Ang-1), stimulating the PI3K/Akt signaling pathway by targeting PIK3R2, and in turn accelerating the wound healing *in vivo* (Zhang et al., 2021).

BMSC-Exos promote angiogenesis while maintaining cardiac function. BMSC-Exos have been widely used in the treatment of acute myocardial infarction and ischemic heart failure. Teng et al. (2015) found that BMSC-Exos could significantly enhance the tubular formation of umbilical vein endothelial cells, inhibit the proliferation of cells *in vitro*, damage the function of T cells, reduce the infarct area, retain the systolic and diastolic function of the heart, increase the density of new functional capillaries, and promote the recovery of blood flow, thus improving the cardiac function after ischemic injury in a rat myocardial infarction model. Moreover, BMSC-Exos contain a high level of miR-29c, cells under hypoxia/reoxygenation, which can inhibit excessive autophagy through PTEN/AKT/mTOR signal pathway and change the expression level of miR-29c, thus reducing myocardial ischemia-reperfusion injury (Li T. et al., 2020). In addition, Chen Q. et al. (2020) showed that miR-125b carried by BMSC-Exos have a certain therapeutic effect on myocardial ischemia-reperfusion injury in rats by improving the survival rate of myocardial cells after myocardial ischemia-reperfusion injury, reducing the rate of apoptosis, alleviating the pathological injury of myocardial tissue, and protecting the myocardium and maintaining cardiac function.

At present, BMSC-Exos has become a focal research area in the treatment of myocardial ischemia-reperfusion injury and heart repair. Differential gradient centrifugation has been used to isolate exosomes derived from BMSCs. Under pressure overload state, BMSC-Exos can protect cardiac muscle, avoid interference caused by cardiac hypertrophy, reduce myocardial cell apoptosis, promote premature senescence of fibroblasts, enhance the anti-fibrosis ability of the heart and protect cardiac function (Chen F. et al., 2020). BMSC-Exos with high expression of macrophage migration inhibitory factor (MIF) has a protective effect on the heart of rats with myocardial infarction. BMSC-Exos and MIF-BMSC-Exos were injected into the infarcted area, respectively; echocardiography was used to evaluate the cardiac function of rats. It was found that both exosomes could significantly restore cardiac function (Liu X. et al., 2020); however, the injection of MIF-BMSC-Exos can better enhance cardiac function, reduce mitochondrial fragmentation and apoptosis of cardiomyocytes. These data suggest that MIF-BMSC-Exos may become a new mechanism for the treatment of myocardial infarction death, and provide a new strategy for the treatment of cardiovascular diseases (Figure 2B).

### Effects of Bone Marrow Mesenchymal Stem Cells-Exos on Lung, Liver, and Kidney

BMSCs have shown promising results in the treatment of acute lung injury (Chang et al., 2009). Liu et al. (2019) found that intravenous injection of BMSC-Exos into the rat model of acute lung injury could down-regulate TLR4 and NF-κB, inhibit TLR4/NF-κB and alleviate acute lung injury induced by ischemia-reperfusion by reducing apoptosis and inflammation. Similarly, Mao et al. (2021) discovered that continuous intravenous injection of BMSC-Exos for 5 days in mice with acute lung injury could inhibit cells apoptosis and promote the expression and relocation of connexin, restoring the barrier function of alveolar epithelial cells and protecting against pulmonary edema.

As a drug carrier, BMSC-Exos have a potential application prospect, especially in the treatment of hepatocellular carcinoma. BMSC-Exos carrying anticancer drug norcantharidin can repair damaged liver tissue in liver sections (Liang et al., 2021). In addition, BMSC-Exos can upregulate the expression of autophagy marker proteins LC3 and Beclin-1, promote the formation of autophagosomes, reduce hepatocyte apoptosis after acute liver failure, and exert a protective effect on the liver (Zhao et al., 2019). BMSCs can also reverse renal injury through exosomes. BMSC-Exos can down-regulate the expression of Sema3A by transporting miR-199a-3p to renal cells, thus activating AKT and ERK pathways, protecting kidneys, and alleviating renal injury (Zhu et al., 2019). Similarly, BMSCs can also minimize renal injury induced by gentamicin through RNA carried by exosomes microvesicles (Reis et al., 2012). These also provide new treatments for kidney disease (Figure 2D).

### Effects of Bone Marrow Mesenchymal Stem Cells-Exos on Tendon and Skin

BMSC-Exos have been suggested as new biological regeneration therapeutic agents for tissue injury. BMSC-Exos can promote the proliferation, migration, and tendon differentiation of tendon stem/progenitor cells (TSPCs). BMSC-Exos embedded in fibrin injected into the patellar tendon defect area of rats showed that exosomes could be released by fibrin, retained in the defect area, and internalized by TSPC. Therefore, BMSC-Exos embedded in fibrin could significantly improve the histological score, enhance the expression of tenomodulin and type I collagen (Col I) and the mechanical properties of the new tendon, and promote the proliferation of local TSPC *in vivo* (Yu et al., 2020). This confirmed the beneficial role of BMSC-Exos in tendon regeneration and provided a potential application prospect for BMSC-Exos in the treatment of tendon injury.

BMSC-Exos have a positive role in wound healing. Skin regeneration is a complex and dynamic process. A full-thickness skin wound model was used to evaluate the effect of BMSC-Exos on skin wound healing cells. It was found that human BMSC-Exos could effectively promote the proliferation of human keratinocytes and human dermal fibroblasts, which accelerated the healing of skin wounds. At the same time, exosomes down-regulated the expression of transforming growth factor β1 (TGF- β1), Smad2, Smad3, and Smad4, and up-regulated TGF-β3 and Smad7 in the TGF-β/Smad signal pathway (Jiang et al., 2020). Furthermore, Wu D. et al. (2020) used magnetic nanoparticles and a constant magnetic field to stimulate BMSCs to construct a new type of exosomes (MAG-BMSC-Exos). Then, the wound healing model was established by scratch test, air hole test, and tube formation experiment. It was found that MAG-BMSC-Exos could promote cell proliferation, migration, and angiogenesis. Local transplantation of MAG-BMSC-Exos could accelerate wound healing, reduce the width of scar and promote angiogenesis. It is worth noting that BMSC-Exos, which were injected into the back skin, revealed a good potential therapeutic effect on alleviating skin ulceration and that the wound healing rate in the exosomes injection group was significantly quicker 2 weeks after operation (Ding et al., 2019). Therefore, BMSC-Exos represents a new therapeutic approach for the clinical treatment of diabetic complications and the treatment of skin trauma (Figures 2E,F).

## Regulation of Bone Marrow Mesenchymal Stem Cells by Exosomes From Extra-Skeletal Organs

### Regulation of Bone Marrow Mesenchymal Stem Cells by Exosomes From Extra-Skeletal Organs Through Factors

**FIGURE 3:**
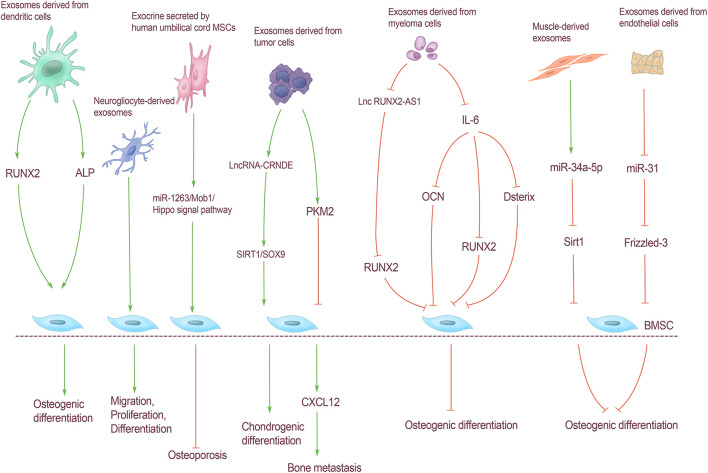
Regulation of BMSCs by exosomes from extra-skeletal organs. Exosomes derived from dendritic cells increased Runx2 expression and ALP activity, and promoted osteogenic differentiation of BMSCs. Neurogliocyte-derived exosomes can promote the migration, proliferation and differentiation of BMSCs. Exosomes secreted by human umbilical cord MSCs can reduce the apoptosis of BMSCs through the miR-1263/Mob1/Hippo signal pathway, and can effectively prevent osteoporosis. Exosomes derived from tumor cells lncRNA-CRNDE can regulate the chondrogenic differentiation of BMSC through SIRT1/SOX9. Exosomes derived from tumor cells act on BMSCs through PKM2, resulting in CXCL12 production in BMSCs, thereby promoting bone metastasis of tumor cells; myeloma cells can directly inhibit RUNX2 by releasing exosomes carrying lnc RUNX2-AS1 through paracrine action on BMSCs, thus inhibiting BMSCs osteogenic differentiation. IL-6 carried by exosomes derived from myeloma cells could inhibit bone differentiation of BMSCs by down-regulating the expression of Runx2, OCN, and Osterix. The expression of miR-34a-5p in muscle-derived exosomes was significantly increased, which could reduce Sirt1 expression and inhibit osteogenic differentiation. Exosomes derived from endothelial cells contain miR-31 into bone through blood circulation and act on BMSCs. Osteoblast differentiation was inhibited by inhibiting Frizzled-3 expression.

Exosomes carry a variety of substances, including some bioactive substances such as proteins and lipids that are essential for signal transduction. Liu Z. et al. (2020) added exosomes derived from myeloma cells to BMSCs and found that IL-6 carried by exosomes could inhibit bone differentiation of BMSCs by down-regulating the expression of runt-related transcription factor 2 (Runx2), OCN, and osterix. Dai et al. (2019) found that exosomes of prostate cancer cells act on BMSCs through pyruvate kinase isozyme type M2 (PKM2) and up-regulate the production of CXCL12 in BMSCs in HIF-1α dependent manner, thereby promoting bone metastasis of tumor cells. In addition to tumor cells, Wang et al. (2014) found that exosomes isolated from dendritic cells can promote osteogenic differentiation of BMSCs *in vitro*. Adding exosomes to BMSCs for osteogenic induction can increase the expression level of Runx2 and the activity of ALP. In addition, glial cell-derived exosomes in the peripheral nervous system can promote the migration, proliferation, and differentiation of BMSCs, which can effectively improve the efficacy of titanium alloy scaffolds in bone repair (Wu Z. et al., 2020). Tofino-Vian et al. (2017) found that exosomes of adiposite-derived stem cells (ASCs) can resist the increased expression of IL-6 and matrix metalloproteinases induced by oxidative stress in osteoarthritis, and play an antioxidant and anti-inflammatory role in osteoarthritis. To delay the progression of osteoarthritis (Figure 3).

### Regulation of Bone Marrow Mesenchymal Stem Cells by miRNA and lncRNA in Exosomes of Extra-Skeletal Organs

MiRNA is a class of non-coding small RNAs 17–24 nt in size that mediates post-transcriptional gene silencing by binding to the 3′-untranslated region (UTR) or open reading frame (ORF) of target Mrna (Bartel, 2004). miRNAs carried by exosomes have key roles in cell differentiation, immune response, tumor cell proliferation, and metastasis. miRNAs carried by muscle-derived exosomes can regulate the osteogenic differentiation of BMSCs. Fulzele et al. (2019) found that with aging and oxidative stress, the expression of miR-34a-5p in muscle and muscle-derived exosomes was significantly increased. The overexpression of miR-34a in C2C12 with lentiviral vector showed that the exosomes isolated from these transfected cells targeted bone *in vivo* and induced BMSCs senescence decreased Sirt1 expression and inhibited osteogenic differentiation (Fulzele et al., 2019). Moreover, exosomes secreted by extraosseous tissues regulate bone homeostasis through miRNA, not only from muscle. Also, the exocrine secreted by human umbilical cord MSCs can reduce BMSCs apoptosis through the miR-1263/Mob1/Hippo signal pathway and can effectively prevent osteoporosis in disuse osteoporosis rats (Yang et al., 2020). Weilner et al. (2016) found that senescent endothelial cells can secrete exosomes containing miR-31 into the bone through blood circulation and act on BMSCs, by inhibiting the expression of Frizzled-3 and then inhibiting osteogenic differentiation. Hashimoto et al. (2018) identified eight highly expressed miRNAs from exosomes isolated from prostate cancer cells by gene chip technology, and proved that mir-940 significantly promoted the osteogenic differentiation of hMSCs *in vitro* cell experiments. The exosomes of tumor cells contain much more specific miRNA than those of normal cells (Shao et al., 2016). However, the effect of tumor exosomes on BMSCs through miRNA has not been reported. Therefore, exploring the regulation of miRNA on BMSCs in exosomes of all kinds of tumor cells is helpful to further understand the mechanism of bone metastasis of tumor cells and provide guidance for the prevention and treatment of bone metastasis of tumor cells.

Long non-coding RNAs (lncRNAs) are a kind of non-coding RNAs with a length of more than 200 nt. LncRNA carried by exocrine mainly transmits information between cells through epigenetic modification, regulation of transcriptional expression, mediation of post-transcriptional regulation, and other specific regulatory modes (Peng et al., 2018). Unlike miRNAs, the correlative studies on the regulation of BMSCs by exosomes derived from extra-skeletal organs through lncRNA focus on bone metastasis of tumor cells. Li et al. (2018) found that myeloma cells can directly inhibit Runx2 through paracrine action on BMSCs by releasing exosomes carrying lncRUNX2-AS1, thus inhibiting the osteogenic differentiation of BMSCs. The expression of lncRNA-CRNDE in exosomes isolated from patients with colorectal cancer is increased, and lncRNA-CRNDE may be related to tumor cell metastasis (Liu et al., 2016). In addition, lncRNA-CRNDE can regulate the process of BMSCs chondrogenic differentiation through SIRT1/Sox9 and promote cartilage repair of osteoarthritis (Shi et al., 2021).

The endocrine regulation effect of exosomes derived from extra-skeletal organs on BMSCs is mainly mediated by cytokines, miRNAs, and lncRNAs. Currently, there are no studies on the regulation of BMSCs by carrying circRNAs; thus, exploring the effect of exocrine-carried circRNAs on BMSCs can further clarify the mechanism of exocrine-mediated intercellular information communication (Figure 3).

## Conclusion

Exosomes have emerged as new elements in the field of medical science. Recent studies have shown that BMSC-Exos can regulate the proliferation and differentiation of osteoblasts and osteoclasts through proteins, lipids, and nucleic acids (mRNA, miRNA, lncRNA, etc.), exerting significant therapeutic effects on the cardiovascular system and nervous system, as well as on intervertebral disc degeneration, tendon regeneration, wound repair, cardiovascular system protection, nerve injury treatment, and clinical diagnosis. Exosomes enter target cells through endocytosis to employ a bioactive role, showing a similar ability to BMSCs. At the same time, they have no non-cellular activity and low immunity, which promotes their use in clinical work. Moreover, they can be easily obtained and stored for some time without damaging their biological activity.

However, our research on BMSC-Exos are still far from being thorough. Taking tumor as an example, for a long time, the research on exosomes has focused on the development of specific targeted anti-tumor vaccines. However, whether its anti-tumor effect is to enhance immunity or cause tolerance, promote proliferation or induce apoptosis, its internal mechanism still needs to be deeply explored. BMSC-Exos have a broad application prospect, and has significant therapeutic effects on the cardiovascular system, nervous system and skeletal muscle. If exosomes are used as research targets, they can play a prominent role in tendon regeneration, wound repair, cardiovascular system protection, nerve injury treatment, clinical disease diagnosis and other aspects. It also plays an important role in the occurrence and development of bone metabolic diseases and opens up new ideas for basic research and clinical diagnosis and treatment of skeletal related diseases. The in-depth study of BMSC-Exos in the future is expected to make this approach an ideal treatment that can be better used to safeguard human organs and tissues and reduce the pain induced by various diseases and complications.

## Author Contributions

LZ gave the brief introduction of this article. ZW, YW, and ZZ were responsible for the manuscript writing. LZ and CL revised the manuscript. All authors approved the final version of this manuscript.

## Conflict of Interest

The authors declare that the research was conducted in the absence of any commercial or financial relationships that could be construed as a potential conflict of interest.

## Publisher’s Note

All claims expressed in this article are solely those of the authors and do not necessarily represent those of their affiliated organizations, or those of the publisher, the editors and the reviewers. Any product that may be evaluated in this article, or claim that may be made by its manufacturer, is not guaranteed or endorsed by the publisher.
